# SKA3/PTTG1/c‐MYC signal loop drives the progression of colorectal cancer

**DOI:** 10.1002/ctm2.1730

**Published:** 2024-06-07

**Authors:** Hao Zhang, Zewen Chang, Chunlin Wang, Ziming Yuan, Yunxiao Liu, Yuliuming Wang, Weiyuan Zhang, Yuchen Zhong, Meng Wang, Chaoxia Zou, Qingchao Tang, Hanqing Hu, Guiyu Wang

**Affiliations:** ^1^ Department of Colorectal Surgery the Second Affiliated Hospital of Harbin Medical University Harbin China; ^2^ Department of Colorectal Cancer Surgery Cancer Hospital of the University of Chinese Academy of Sciences (Zhejiang Cancer Hospital) Hangzhou China; ^3^ Department of Biochemistry and Molecular Biology of Harbin Medical University Harbin China

To the editor

Colorectal cancer (CRC) represents a prominent neoplasm globally ranking third in prevalence amongst humans and standing as the second leading cause for cancer‐associated fatalities.^1^, ^2^ The fundamental molecular mechanisms driving CRC progress remain largely uninvestigated. Spindle and kinetochore‐associated complex subunit 3 (SKA3) is an indispensable constituent of the SKA complex that plays a crucial role in stabilising the interaction between kinetochores and microtubules during mitosis.^3^, ^4^ Emerging evidence suggests SKA3 is implicated in the progression of various malignancies.^5^, ^6^ However, the functional role and mechanism of SKA3 in CRC remain unclear. Here, we demonstrated that SKA3 is pivotal in CRC progress and the SKA3/PTTG1/c‐MYC axis could potentially be a latent therapeutic target for CRC.

We initially conducted RNA‐seq analysis using five pairs of CRC and normal tissues and found that SKA3 was significantly upregulated in CRC tissues (Figure S1A), which was also validated in The Cancer Genome Atlas (TCGA) and Gene Expression Omnibus (GEO) databases (GSE83889, GSE106582, and GSE87211) (Figure S1B–E). A Kaplan–Meier analysis in GSE29623 demonstrated patients with high expression levels of SKA3 had unfavorable overall survival (Figure S1F). Western blot assay and immunohistochemical staining (IHC) also confirmed a significant increase of the SKA3 expression in CRC tissues (Figure 1A,B). Compared to normal colorectal cell CCD‐18Co, CRC cells displayed elevated levels of SKA3 expression (Figure S1G). In vitro, SKA3 knockdown reduced proliferation, migration and invasion of HCT116 and RKO cells (Figure 1C–G and Figure S2A), whilst SKA3 overexpression leaded to opposite effects (Figure S2A–H). Besides, SKA3 knockdown diminished Proliferating Cell Nuclear Antigen (PCNA) expression, whilst SKA3 overexpression resulted in increased PCNA expression (Figure 1H and Figure S2I). According to the western blot assay of Epithelial Mesenchymal Transition (EMT)‐associated markers (E‐cadherin and N‐cadherin), SKA3 could also induce the EMT process (Figure 1H and Figure S2I). In vivo, both the volume and weight of xenograft tumours in the shSKA3 group exhibited significant delays (Figure 1I–K). The IHC analysis demonstrated a significant decrease in Ki‐67 expression, N‐cadherin and vimentin, but an increase in E‐cadherin within the shSKA3 group (Figure 1L). Besides, the shSKA3 group showed a marked decrease in both the number of lung metastatic nodules and lung weight compared to the control group (Figure 1M,N).

**FIGURE 1:**
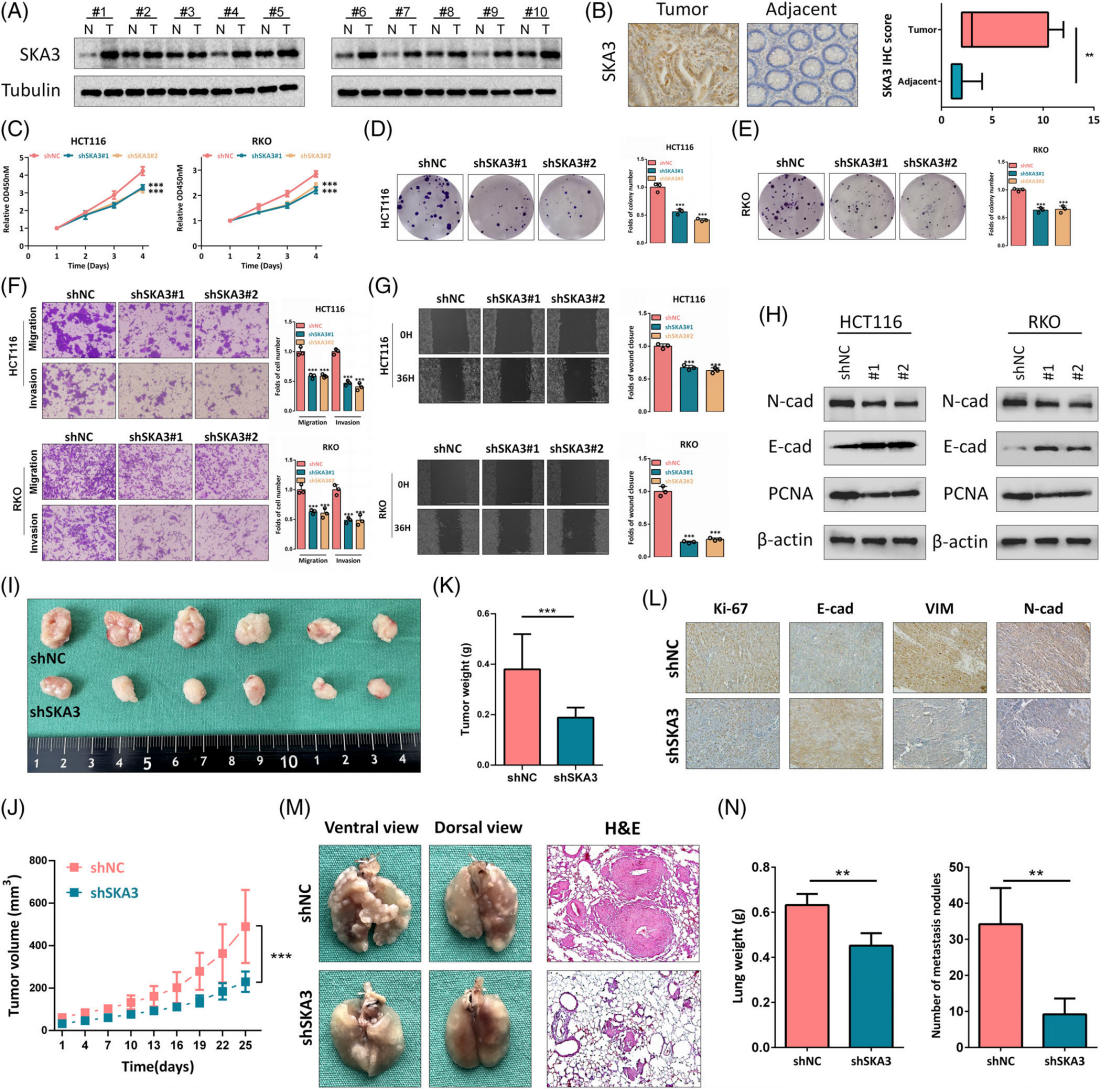
SKA3 promotes proliferation and metastasis of colorectal cancer. (A) The expression levels of SKA3 were assessed in 10 pairs of clinical colorectal cancer (CRC) samples and their corresponding adjacent tissues using western blot analysis. (B) Representative images of IHC in 13 pairs of CRC and adjacent tissues and IHC staining for SKA3. (C) Cell Counting Kit 8 (CCK8) assay indicates the change in cell viability of HCT116 and RKO cells under SKA3 knockdown. (D,E) Representative images and quantification of colony formation assay depicting the growth of HCT116 and RKO cells under SKA3 knockdown. (F) Representative images and quantification of transwell assay depicting the migration and invasion abilities of HCT116 and RKO cells under SKA3 knockdown. (G) Representative images and quantification of wound healing assay depicting the migration ability of HCT116 and RKO cells under SKA3 knockdown. (H) Western blotting was used to measure the expression levels of PCNA and EMT‐related markers in HCT116 and RKO cells with SKA3 knockdown or overexpression. (I,J) Tumour volumes in the shNC and shSKA3 groups were measured every 3 days and growth curves were plotted (*n* = 6 per group). (K) Tumour weights of shNC and shSKA3 groups in the xenograft model (*n* = 6 per group). (L) Relative expression level and representative IHC staining images of Ki67, E‐cadherin, N‐cadherin and Vimentin for indicated tumour tissues. (M) Representative lung images and H&E staining analysis of mouse lung sections showing tumour lesions (*n* = 5 per group). (N) The number of lung metastatic nodules and lung weight were counted (*n* = 5 per group). SKA3, Spindle and kinetochore‐associated complex subunit 3; IHC, immunohistochemical.

Then, we performed RNA‐seq analysis in shNC and shSKA3 HCT116 cells and the Gene Set Enrichment Analysis (GSEA) showed a notable positive correlation between SKA3 and MYC‐targeted signalling pathways (Figure 2A–C), which was also supported by the TCGA database (Figure 2D and Figure S3A,B). Additionally, the IHC results showed dxenograft tumours in the shSKA3 group exhibited lower levels of c‐MYC protein (Figure 2E). The experimental results indicated that when SKA3 was knocked down, both protein and mRNA levels of c‐MYC decreased (Figure 2F). Conversely, overexpression of SKA3 led to an increased expression level of c‐MYC (Figure 2G). Besides, SKA3 and c‐MYC target gene mRNA levels were positively corelated in the TCGA database and GEO database (GSE83889, GSE106582 and GSE87211) (Figure 2H), and it was further confirmed by the qPCR analysis (Figure 2I,J). Subsequently, to ascertain whether the effects of SKA3 on CRC cell were dependent on c‐MYC, rescue experiments were conducted and the efficacy of interference was confirmed through western blot analysis (Figure S3C). The results demonstrated that the increased abilities of cell proliferation, migration and invasion induced by SKA3 were abolished by knocking down c‐MYC (Figure 2K–N and Figure S3D–G).

**FIGURE 2:**
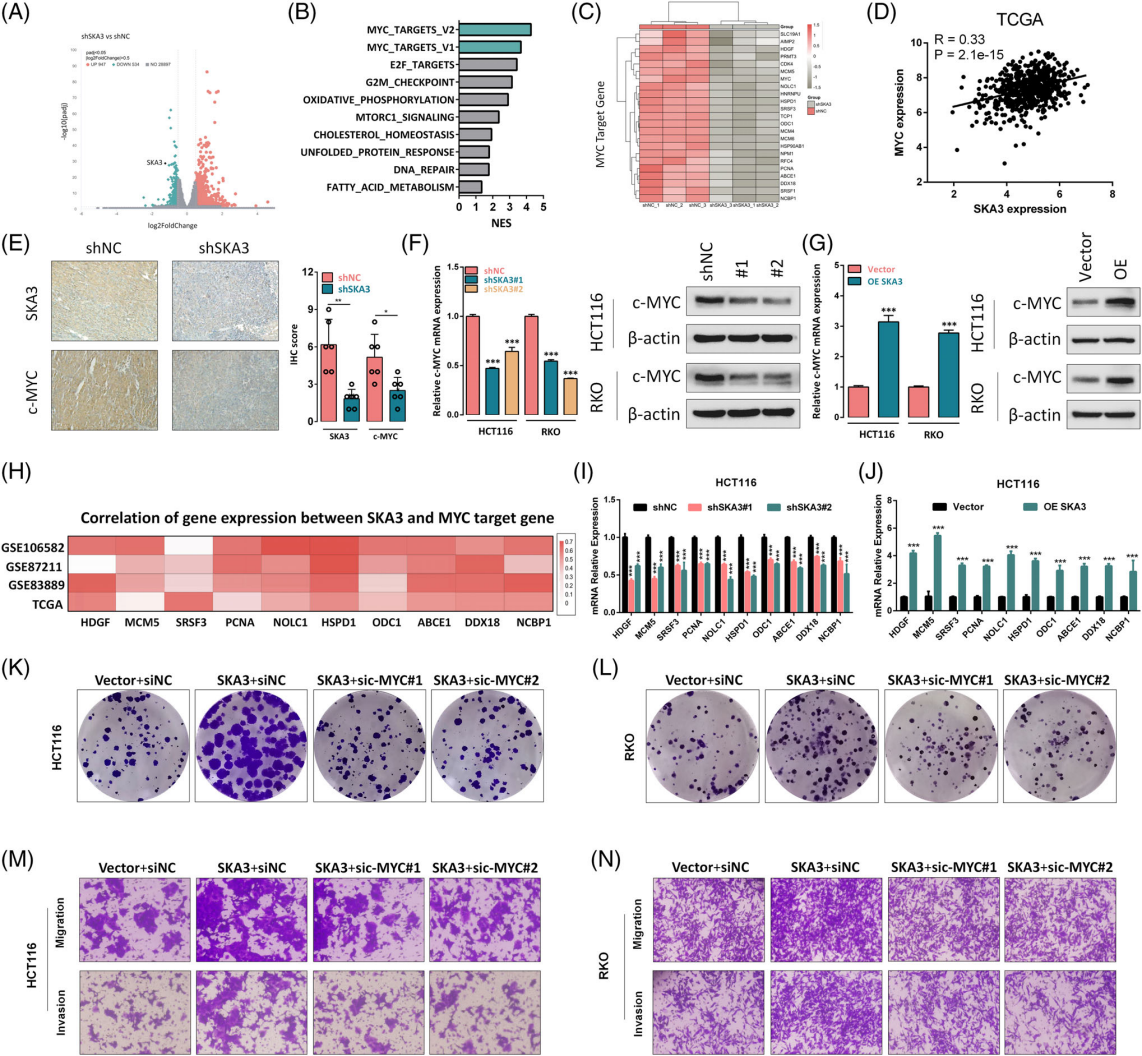
SKA3 is positively related to c‐MYC expression and MYC‐targeted signalling pathways. (A) Volcano plot of all expressed genes was shown to identify up‐ or downregulated transcripts obtained from RNA‐seq data in shSKA3 HCT116 cell. (B) Gene set enrichment analysis (GSEA) in shSKA3 HCT116 cells using the oncogenic signatures gene set. (C) Heatmap analysis of genes with significant changes in the MYC target gene set upon SKA3 knockdown in HCT116 cells. (D) The correlation between the mRNA levels of SKA3 and c‐MYC in the TCGA database. (E) Protein levels of SKA3 and c‐MYC in the subcutaneous tumour tissues of shNC and shSKA3 groups were detected by IHC. (F,G) c‐MYC expression was detected by q‐PCR and western blotting after SKA3 knockdown in HCT116 and RKO cells. (H) Correlation of gene expression between SKA3 and MYC target gene in TCGA and GEO database. (I) mRNA level of MYC target gene was detected by q‐PCR after SKA3 knockdown in HCT116. (J) mRNA level of MYC target gene was detected by q‐PCR after SKA3 overexpression in HCT116. (K,L) Colony formation assay indicated the change in cell proliferation ability of CRC cells under SKA3 overexpression was depend on c‐MYC. (M,N) Transwell assay indicated the change in cell migration and invasion abilities of CRC cells under SKA3 overexpression was depend on c‐MYC. SKA3, Spindle and kinetochore‐associated complex subunit 3; CRC, colorectal cancer.

To further investigate the regulatory mechanism of SKA3 on c‐MYC, we employed the BioGRID and STRING online databases to identify potential protein interactors of SKA3 (Figure 3A). Amongst these candidates, PTTG1 caught our attention as it functions as a transcriptional activator for c‐MYC.^7^ Chromatin Immunoprecipitation (ChIP) assay demonstrated direct binding between PTTG1 and the promoter region of c‐MYC in CRC cells (Figure 3B,C). Dual luciferase reporter assay showed that the reduction of the PTTG1 expression resulted in a notable decreased luciferase activity of the c‐MYC‐promoter‐WT reporter in HCT116 cell, whilst the luciferase activity of MUT c‐MYC reporter did not show any significant change (Figure 3D). Additionally, knockdown of PTTG1 in HCT116 and RKO cells resulted in a notable decrease in both c‐MYC mRNA and protein levels (Figure 3E and Figure S4A,B). Therefore, PTTG1 is a transcriptional activator for c‐MYC in CRC cells (Figure 3F). As expected, co‐immunofluorescence analysis and co‐immunoprecipitation (Co‐IP) assay confirmed the interaction between SKA3 and PTTG1 (Figure 3G,H). Intriguingly, our findings demonstrated although SKA3 could not significantly alter the PTTG1 protein expression level (Figure S4C,D), SKA3 overexpression effectively facilitated the nuclear translocation of PTTG1 (Figure 3I–M). The dual‐luciferase analysis revealed a significant reduction in the c‐MYC transcriptional activity upon knockdown of SKA3 in CRC cells, whilst overexpression of SKAS yielded an opposite effect (Figure 3N,O). However, it was observed that SKA3 had no influence on the activity of the c‐MYC promoter containing mutant PTTG1 binding sites (Figure 3N,O). These results provide evidence that supports the positive role of SKA3 in regulating c‐MYC transcriptional activity through its interaction with PTTG1 and facilitation of PTTG1 nuclear translocation.

**FIGURE 3:**
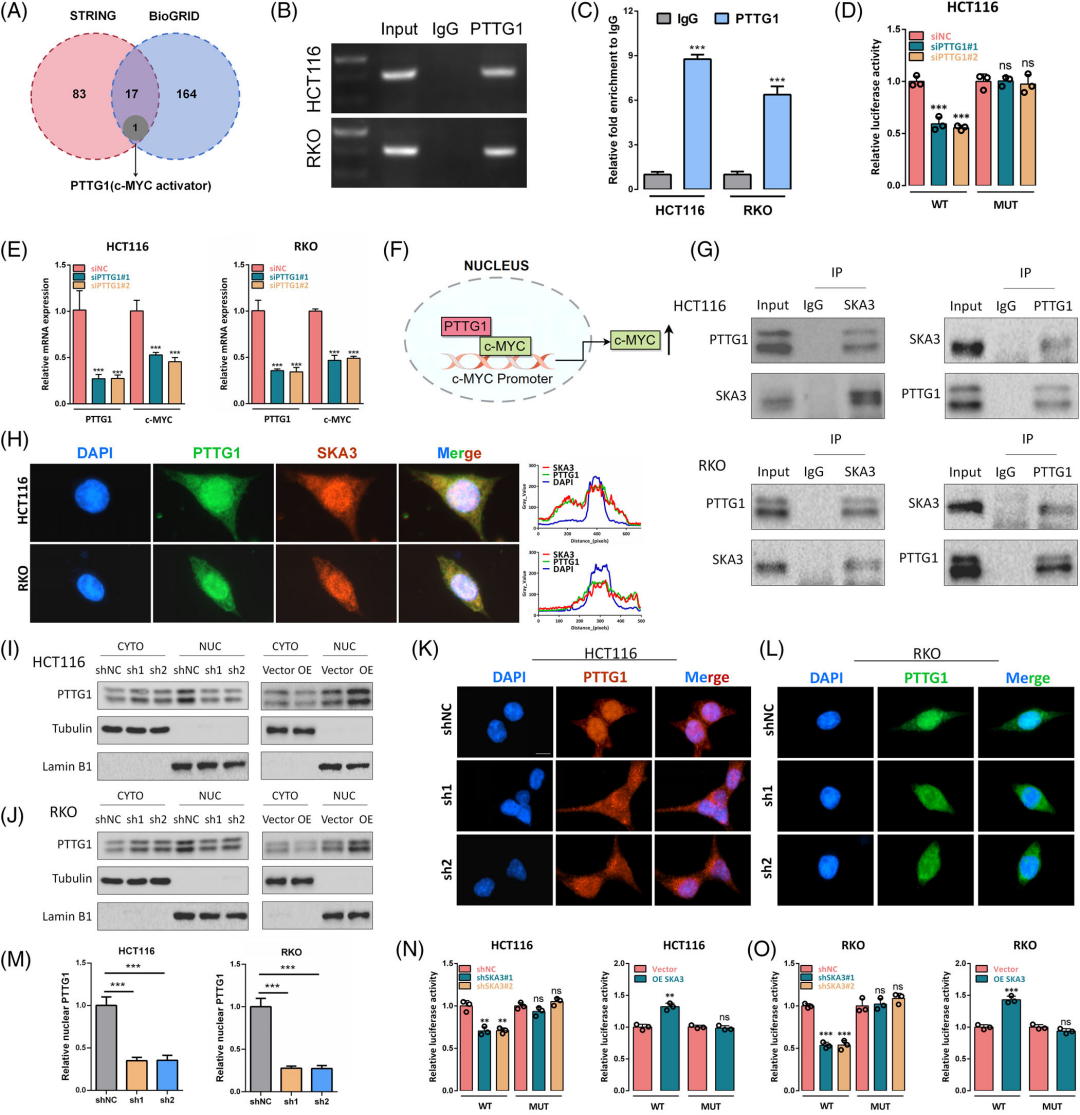
SKA3 could interact with PTTG1 and facilitate nuclear translocation of PTTG1. (A) The Venn diagram was used to illustrate the overlap between proteins interacting with SKA3 analysed by STRING and BioGRID database. (B,C) PTTG1 enrichment at the c‐MYC promoter was measured using a PTTG1 antibody to perform the ChIP assay. (D) The dual‐luciferase analysis for c‐MYC transcriptional activity was performed in HCT116 cell with or without PTTG1 knockdown. (E) c‐MYC expression was detected by q‐PCR after PTTG1 knockdown in HCT116 and RKO cells. (F) Schematic diagram showing that PTTG1 is a transcriptional activator for c‐MYC in CRC cells. (G) Co‐immunoprecipitation analysis determined the interaction between SKA3 and PTTG1 in HCT116 and RKO cells. (H) Co‐immunofluorescence was performed for SKA3 (red) and PTTG1 (green) in HCT116 and RKO cells (scale bar 10 µm). (I,J) Western blot analysis to show the PTTG1 expression in the nucleus and cytoplasm of HCT116 and RKO cells with SKA3 knockdown or overexpression. (K,L) Immunofluorescence showing the subcellular distribution of PTTG1 in HCT116 and RKO cells with SKA3 knockdown. (M) Histograms show densitometric analysis of nuclear PTTG1. (N,O) The dual‐luciferase analysis for c‐MYC transcriptional activity was performed in HCT116 and RKO cells with SKA3 knockdown or overexpression. SKA3, Spindle and kinetochore‐associated complex subunit 3; CRC, colorectal cancer.

Moreover, the Cistrome DB Toolkit and JASPAR analysis revealed that c‐MYC may exert regulatory effects on the SKA3 expression as a transcription factor and the c‐MYC binding site (TCACCTGC) was predicted to be situated at −167 to −174 upstream of the SKA3 TSS (Figure 4A and Figure S5). The GRNdb database and single‐cell RNA sequencing also confirmed a significant correlation between SKA3 expression and c‐MYC expression at the transcriptional level (Figure 4B). In HCT116 and RKO cells, c‐MYC knockdown could result in a decrease in SKA3 mRNA and protein expression (Figure 4C–E). Besides, the luciferase reporter and ChIP assays indicated c‐MYC could bind to the SKA3 promoter and enhance SKA3 transcription (Figure 4F–I). IHC staining on CRC tissue samples also revealed a significant correlation between expression levels of SKA3 and c‐MYC (Figure 4J).

**FIGURE 4:**
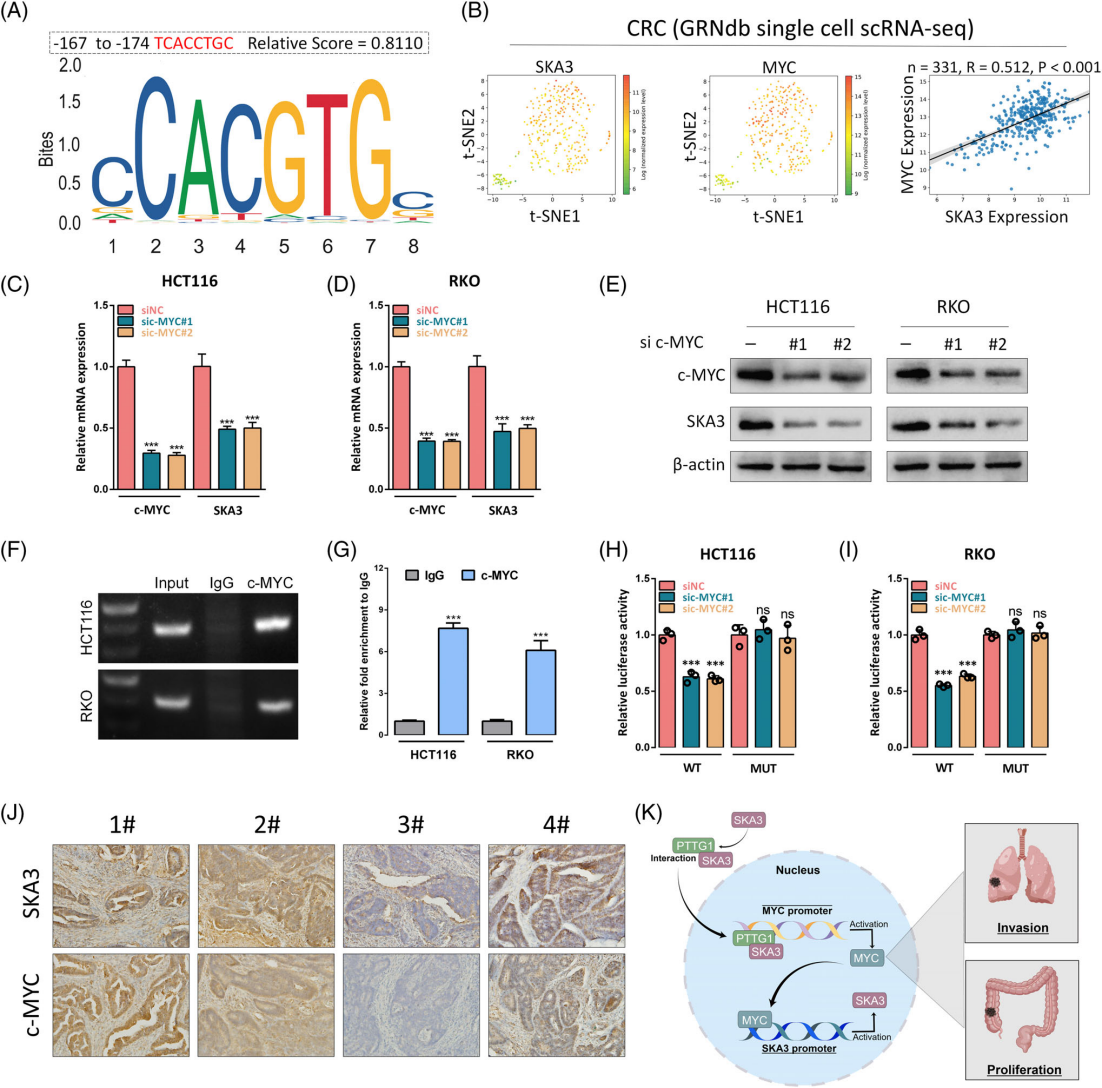
c‐MYC promotes SKA3 expression as a transcription factor. (A) Analysis using the JASPAR database shows the predicted c‐MYC binding motifs in the SKA3 promoter. (B) Feature plots and a scatterplot depict the correlation between SKA3 and c‐MYC in the TCGA‐CRC single cell transcriptome. (C–E) SKA3 expression was detected by q‐PCR and western blotting after c‐MYC knockdown in HCT116 and RKO cells. (F,G) c‐MYC enrichment at the SKA3 promoter was measured using a c‐MYC antibody to perform the ChIP assay. (H,I) The dual‐luciferase analysis for SKA3 transcriptional activity was performed in HCT116 and RKO cells with or without c‐MYC knockdown. (J) Representative IHC staining of CRC tissues showed the correlation between c‐MYC and SKA3 expression levels. (K) Schematic representation of a model for the major molecular mechanisms of “SKA3–PTTG1–c‐MYC” signal loop which promotes the proliferation and metastasis of colorectal cancer. SKA3, Spindle and kinetochore‐associated complex subunit 3; CRC, colorectal cancer; IHC, immunohistochemical.

In summary, we found SKA3 could interact with PTTG1 (a transcriptional activator for c‐MYC), which facilitates nuclear translocation of PTTG1, resulting in upregulation of c‐MYC expression. Moreover, the SKA3 expression could be transcriptionally upregulated by c‐MYC, resulting in a positive feedback axis to promote CRC progression (Figure 4K).

## AUTHOR CONTRIBUTIONS

*Conception and design*: Hao Zhang, Qingchao Tang, Hanqing Hu, Guiyu Wang. *Experiment performance and data acquisition*: Hao Zhang, Zewen Chang. *Analysis and interpretation of data*: Chunlin Wang, Ziming Yuan, Yunxiao Liu, Yuliuming Wang, Weiyuan Zhang, Yuchen Zhong, Meng Wang, Chaoxia Zou. *Writing, review, and/or revision of the manuscript*: Hao Zhang, Zewen Chang, Qingchao Tang, Hanqing Hu, Guiyu Wang.

## CONFLICT OF INTEREST STATEMENT

The authors declare no conflicts of interest.

## FUNDING INFORMATION

Supported by the National Natural Science Foundation of China, Grant numbers: 82103030, 62276084, U23A20482.

## ETHICS STATEMENT

The experiments were performed according to the approved guidelines established by the Institutional Review Board at the Second Affiliated Hospital of Harbin Medical University, Harbin, China. Animal experiments were performed according to procedures approved by the Institutional Animal Care and Use Committee at the Second Affiliated Hospital of Harbin Medical University, Harbin, China.

## Supporting information


**Figure S1 (**A) RNA‐seq analysis on five pairs of CRC and normal tissues revealed SKA3 was significantly upregulated in CRC tissues compared to normal tissues (display of partial differential genes). (B) Expression of SKA3 in CRC and nontumour tissues in the TCGA dataset. (C–E) Expression of SKA3 in CRC and nontumour tissues in the GSE83889, GSE106582 and GSE87211 datasets. (F) High SKA3 levels predict worse OS based on the GSE29623 dataset. Median expression level was used to stratify SKA3 High and SKA3 low groups to analyse OS. (G) A Western blot analysis was employed to quantify the protein expression levels of SKA3 in seven human colorectal cancer (CRC cell lines, as well as the normal colon cell line CCD‐18Co.

**Figure S2** (A) The expression of SKA3 was determined by western blotting in HCT116 and RKO cells after knockdown or overexpression of SKA3. (B,C) CCK8 assay indicates the change in cell viability of HCT116 and RKO cells under SKA3 overexpression. (D,E) Representative images and quantification of colony formation assay depicting the growth of HCT116 and RKO cells under SKA3 overexpression. (F,G) Representative images and quantification of wound healing assay depicting the migration ability of HCT116 and RKO cells under SKA3 overexpression. (H) Representative images and quantification of transwell assay depicting the migration and invasion abilities of HCT116 and RKO cells under SKA3 overexpression. (I) Western blotting was used to measure the expression levels of PCNA and EMT‐related markers in HCT116 and RKO cells with SKA3 overexpression.

**Figure S3** (A,B) GSEA analysis suggested that SKA3 is related to MYC‐targeted signalling pathways in CRC using TCGA datasets. (C) sic‐MYC or siNC was transfected into HCT116 and RKO cells overexpressing SKA3, and the efficacy of interference was confirmed through western blot analysis. (D,E) CCK8 assay indicated the change in cell proliferation ability of CRC cells under SKA3 overexpression was depend on c‐MYC. (F) Western blotting was used to measure the expression levels of PCNA and EMT‐related markers in HCT116 and RKO cells in the rescue experiment. (G) Wound healing assay indicated the change in cell migration ability of CRC cells under SKA3 overexpression was depend on c‐MYC.

**Figure S4** (A,B) c‐MYC expression was detected by western blotting after PTTG1 knockdown in HCT116 and RKO cells. (C,D) PTTG1 expression was detected by western blotting in HCT116 and RKO cells with SKA3 knockdown or over expression.

**Figure S5** Transcription factor with high regulatory potential for SKA3 (10 k distance to TSS) in the Cistrome DB Toolkit.

## Data Availability

The data which was used and/or analyzed during this study is available from the corresponding author on reasonable request.
