# Supplementary figures and images for: SKA3/PTTG1/c‐MYC signal loop drives the progression of colorectal cancer
Source: Clin Transl Med. 2024 Jun 7;14(6):e1730. doi: 10.1002/ctm2.1730 (PMC11161389; doi:10.1002/ctm2.1730)

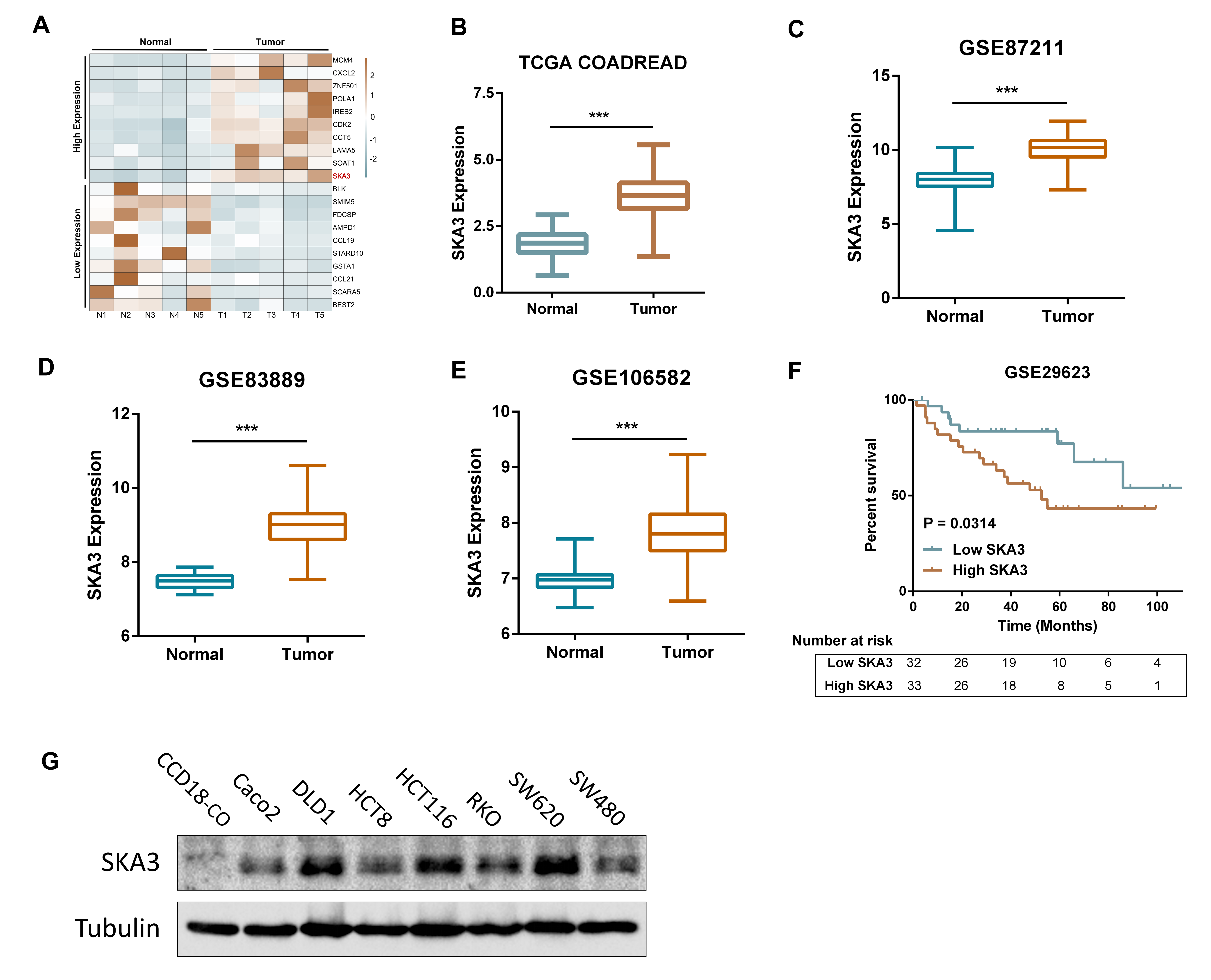

Supplement: Supplementary file 1 — Figure S1 (A) RNA‐seq analysis on five pairs of CRC and normal tissues revealed SKA3 was significantly upregulated in CRC tissues compared to normal tissues (display of partial differential genes). (B) Expression of SKA3 in CRC and nontumour tissues in the TCGA dataset. (C–E) Expression of SKA3 in CRC and nontumour tissues in the GSE83889, GSE106582 and GSE87211 datasets. (F) High SKA3 levels predict worse OS based on the GSE29623 dataset. Median expression level was used to stratify SKA3 High and SKA3 low groups to analyse OS. (G) A Western blot analysis was employed to quantify the protein expression levels of SKA3 in seven human colorectal cancer (CRC cell lines, as well as the normal colon cell line CCD‐18Co. [file CTM2-14-e1730-s004.png]

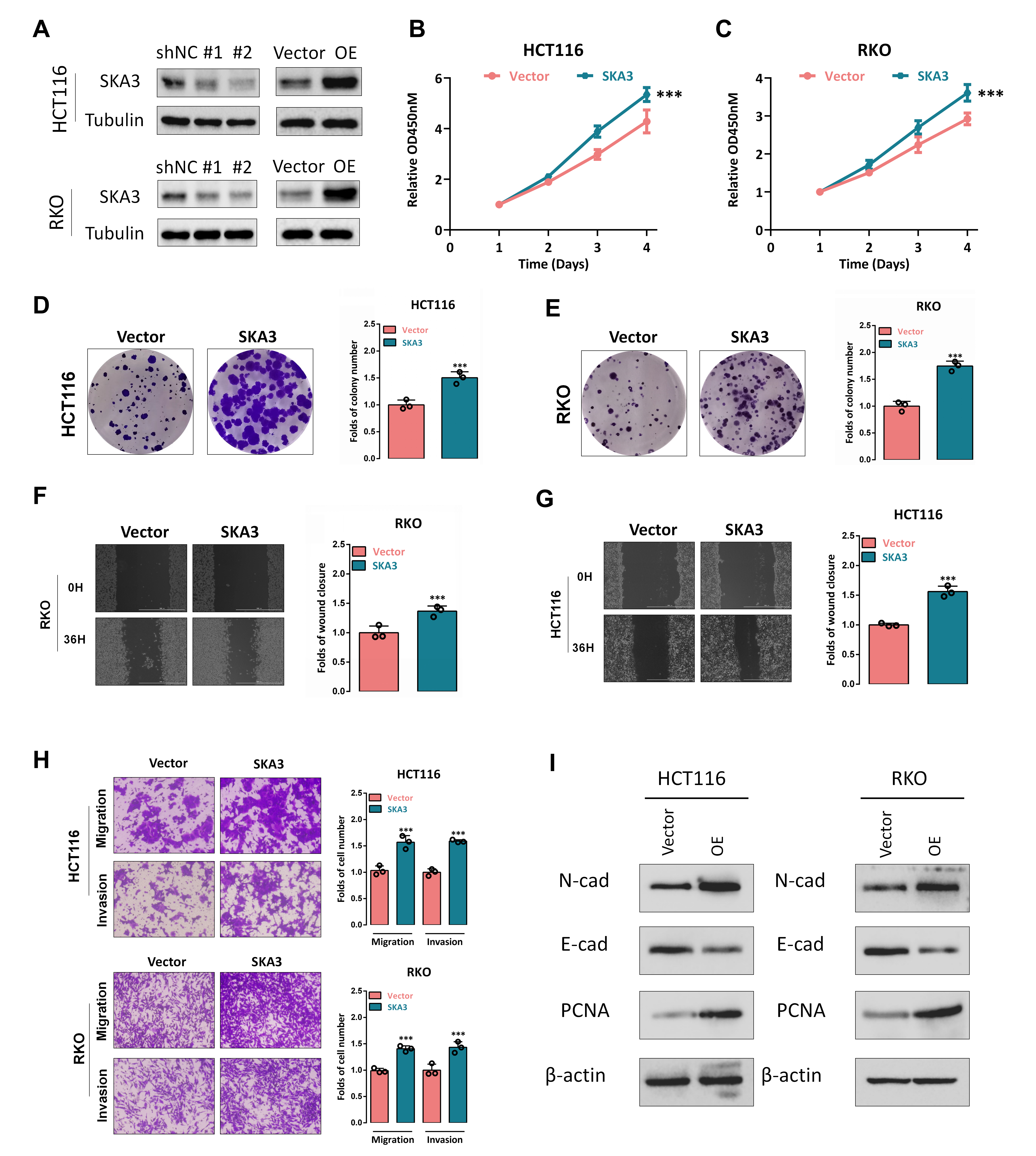

Supplement: Supplementary file 2 — Figure S2 (A) The expression of SKA3 was determined by western blotting in HCT116 and RKO cells after knockdown or overexpression of SKA3. (B,C) CCK8 assay indicates the change in cell viability of HCT116 and RKO cells under SKA3 overexpression. (D,E) Representative images and quantification of colony formation assay depicting the growth of HCT116 and RKO cells under SKA3 overexpression. (F,G) Representative images and quantification of wound healing assay depicting the migration ability of HCT116 and RKO cells under SKA3 overexpression. (H) Representative images and quantification of transwell assay depicting the migration and invasion abilities of HCT116 and RKO cells under SKA3 overexpression. (I) Western blotting was used to measure the expression levels of PCNA and EMT‐related markers in HCT116 and RKO cells with SKA3 overexpression. [file CTM2-14-e1730-s003.png]

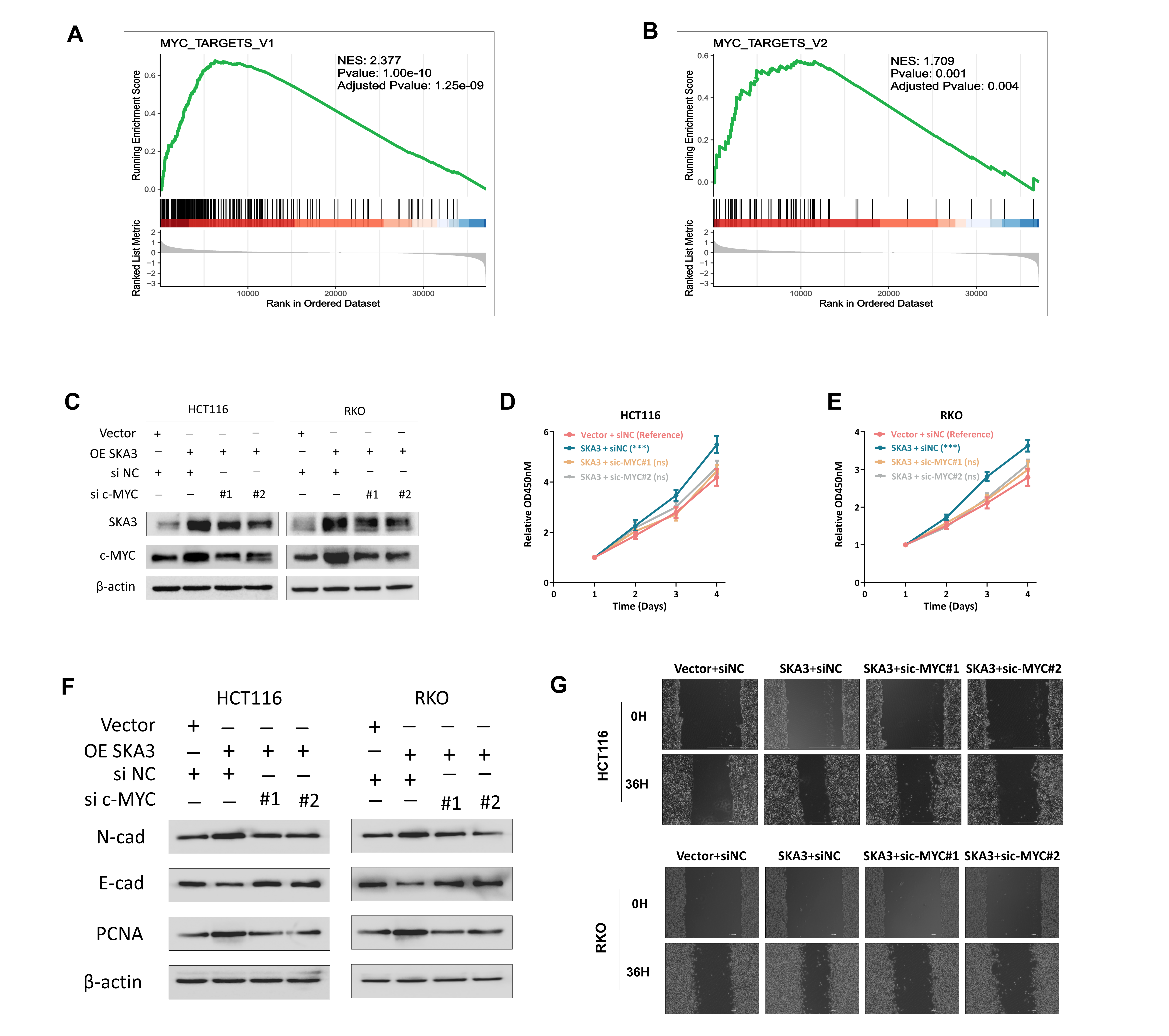

Supplement: Supplementary file 3 — Figure S3 (A,B) GSEA analysis suggested that SKA3 is related to MYC‐targeted signalling pathways in CRC using TCGA datasets. (C) sic‐MYC or siNC was transfected into HCT116 and RKO cells overexpressing SKA3, and the efficacy of interference was confirmed through western blot analysis. (D,E) CCK8 assay indicated the change in cell proliferation ability of CRC cells under SKA3 overexpression was depend on c‐MYC. (F) Western blotting was used to measure the expression levels of PCNA and EMT‐related markers in HCT116 and RKO cells in the rescue experiment. (G) Wound healing assay indicated the change in cell migration ability of CRC cells under SKA3 overexpression was depend on c‐MYC. [file CTM2-14-e1730-s002.png]

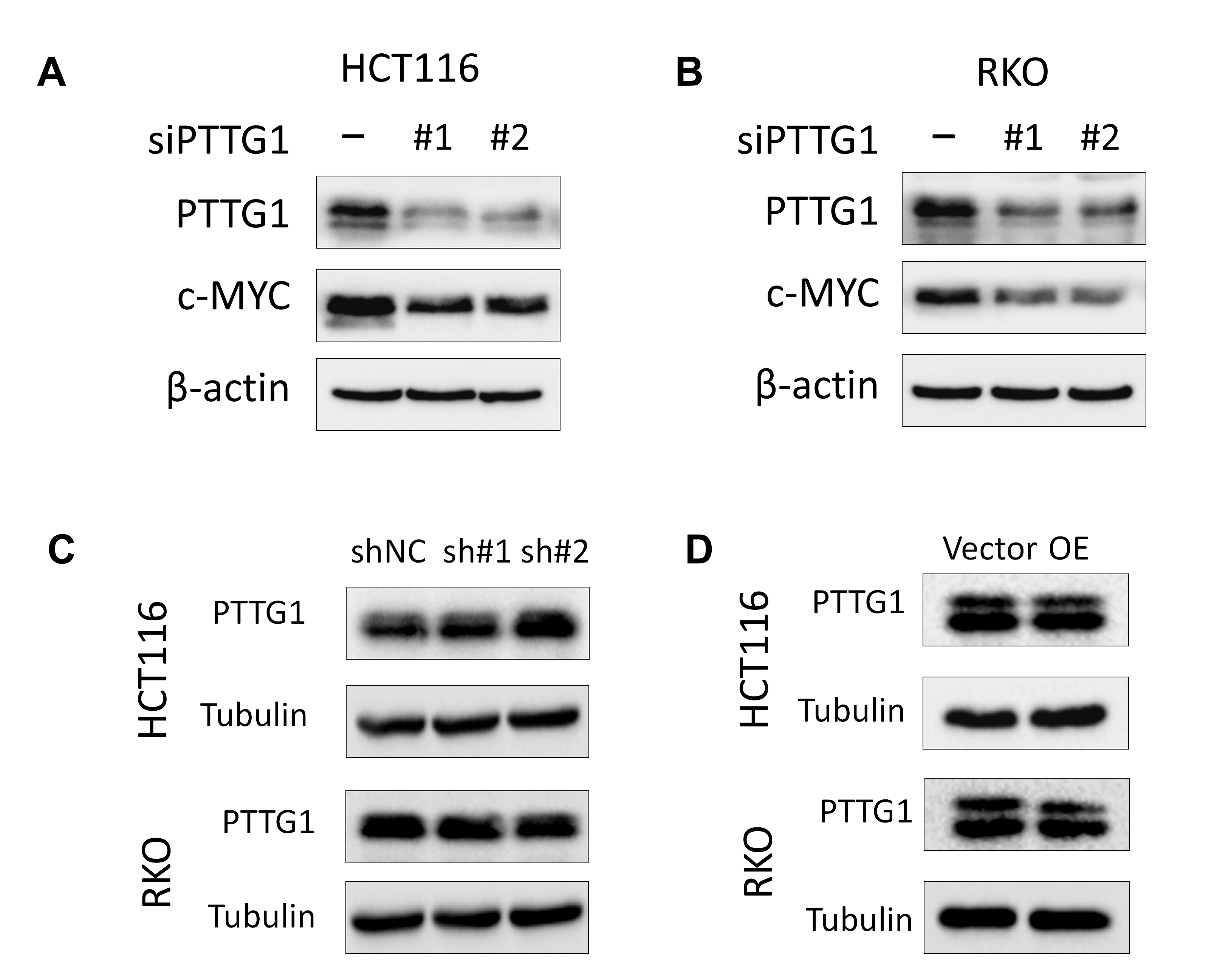

Supplement: Supplementary file 4 — Figure S4 (A,B) c‐MYC expression was detected by western blotting after PTTG1 knockdown in HCT116 and RKO cells. (C,D) PTTG1 expression was detected by western blotting in HCT116 and RKO cells with SKA3 knockdown or over expression. [file CTM2-14-e1730-s005.png]

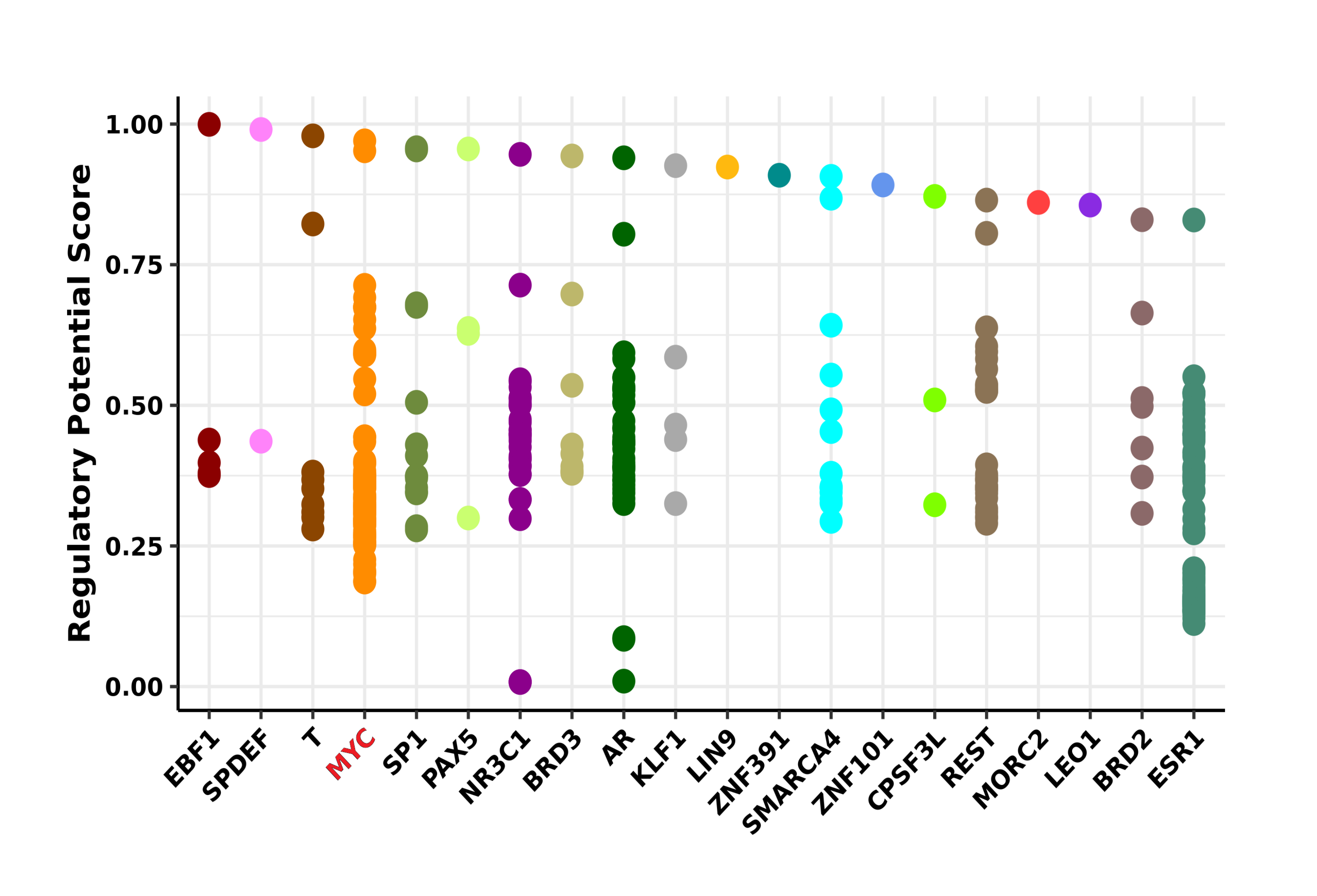

Supplement: Supplementary file 5 — Figure S5 Transcription factor with high regulatory potential for SKA3 (10 k distance to TSS) in the Cistrome DB Toolkit. [file CTM2-14-e1730-s001.png]
